# Increasing Resistance in Commensal *Escherichia coli*, Bolivia and Peru 

**DOI:** 10.3201/eid1402.070138

**Published:** 2008-02

**Authors:** Alessandro Bartoloni, Lucia Pallecchi, Costanza Fiorelli, Tiziana Di Maggio, Connie Fernandez, Ana Liz Villagran, Antonia Mantella, Filippo Bartalesi, Marianne Strohmeyer, Angela Bechini, Herlan Gamboa, Hugo Rodriguez, Charlotte Kristiansson, Göran Kronvall, Eduardo Gotuzzo, Franco Paradisi, Gian Maria Rossolini

**Affiliations:** * Università di Firenze, Florence, Italy; † Università di Siena, Siena, Italy; ‡ Hospital Apoyo Yurimaguas, Yurimaguas-Loreto, Peru; § Servicio Departamental de Salud Santa Cruz, Camiri, Bolivia; ¶ Karolinska Institute, Stockholm, Sweden; # Universidad Peruana Cayetano Heredia, Lima, Peru

**Keywords:** Escherichia coli, antibiotic resistance, commensal bacteria, fluoroquinolones, cephalosporins, Peru, Bolivia, letter

**To the Editor:** The global increase of antimicrobial-drug resistance, including resistance to the new and most potent antimicrobial agents, is a major public health concern. In low-resource countries, where bacterial infections are still among the major causes of death, especially for children, it is of particular concern (*1*).

ANTRES (Towards Controlling Antimicrobial Use and Resistance in Low-income Countries—An Intervention Study in Latin America) is a research project on antimicrobial-drug use and resistance in low-resource countries of Latin America (see www.unifi.it/infdis/antres/default.htm). In 2002, the baseline ANTRES study showed a high rate of fecal carriage of *Escherichia coli* with acquired resistance to several antimicrobial drugs, especially older drugs (e.g., ampicillin, trimethoprim-sulfamethoxazole, tetracycline, streptomycin, and chloramphenicol), in preschool children from 4 urban settings in Bolivia and Peru (*2*). We report the results of a second cross-sectional study, conducted in 2005, that evaluated the evolution of antimicrobial-drug resistance in the studied areas.

We studied healthy children 6–72 months of age from each of 4 urban areas: 2 in Bolivia (Camiri, Santa Cruz Department; Villa Montes, Tarija Department) and 2 in Peru (Yurimaguas, Loreto Department; Moyobamba, San Martin Department). The study design, sampling and inclusion criteria, methods, and ethical issues were the same as those of the baseline study (*2*). The study was carried out over 4 months (September–December 2005), the same seasonal period as in the previous study. No significant differences in sex ratios were found among children enrolled from the different areas, whereas minor differences were found for age. No statistical differences were found between the 2002 baseline study and the 2005 study results in terms of numbers of children (3,193 vs. 3,174) and sex ratios (0.94 vs. 0.95) (Table). Statistical analyses were performed by using Stata 9.0 (Stata Corp., College Station, TX, USA). Logistic regression models were used to compare the antimicrobial-drug resistance rates in 2002 and 2005, considering the combined influences of age, sex, city, and country.

**Table:** Antimicrobial drug–resistance rates of *Escherichia coli* as part of  commensal flora in children, Bolivia and Peru, 2002 and 2005* *Expanded Table available online at www.cdc.gov/EID/content/14/2/338-T.htm.

Drug†	2002	2005	p value‡
AMP	95	96	<0.05
CRO	0.1	1.7	<0.001
TET	93	93	NS
SXT	94	94	NS
CHL	70	69	NS
STR	82	92	<0.001
KAN	28	29	<0.05
GEN	21	27	<0.001
AMK	0.4	0.1	NA
NAL	35	57	<0.001
CIP	18	33	<0.001

Prevalence expressed as percentages. In 2002, n = 3,174, mean age 34.8 mo; in 2005, n = 3,193, mean age 33.7 mo (mean age p<0.05).  †AMP, ampicillin; CRO, ceftriaxone; TET, tetracycline; SXT, trimethoprim-sulfamethoxazole; CHL, chloramphenicol; STR, streptomycin; KAN, kanamycin; GEN, gentamicin; AMK, amikacin; NAL, nalidixic acid; CIP, ciprofloxacin.  ‡Wald test applied to establish the statistical significance of parameters obtained from logistic regression analysis; NS, not significant; NA, not applicable (due to lack of variability of data).

Data from the 2005 survey confirmed high resistance rates for ampicillin, trimethoprim-sulfamethoxazole, tetracycline, streptomycin, and chloramphenicol. The differences in resistance rates observed between 2002 and 2005 for these drugs, although sometimes statistically significant, are probably of limited epidemiologic relevance due to the high rates of antimicrobial-drug resistance found in the *E. coli* population in both surveys. The most relevant finding of the 2005 survey was the remarkable increase since 2002 in the resistance rates to fluoroquinolones and expanded-spectrum cephalosporins (Table). Molecular analysis showed that the dramatic increase in rates of resistance to expanded spectrum cephalosporins was mostly the result of dissemination of CTX-M-type extended-spectrum β-lactamase determinants (*3*). Concerning the association between sex and resistance rates, the higher resistance rates observed for some agents and in some settings for boys in the baseline study were not confirmed, except in 1 case (kanamycin in Camiri, p = 0.04) (*2*). Analysis by age (not performed for amikacin due to low numbers of resistant isolates) confirmed the occurrence of higher resistance rates for the youngest age group and an overall decreasing trend by age for all agents, except ciprofloxacin and gentamicin. For these 2 agents, resistance rates increased, although not significantly (p = 0.95 and p = 0.55, respectively) (*2*). Although we did not specifically address factors potentially involved in this phenomenon, we will address them in future investigations.

Increasing resistance to fluoroquinolones and expanded-spectrum cephalosporins among *E. coli* clinical isolates has been observed in several parts of the world and complicates the management of infections (*4**,**5*). Recently, intestinal colonization with fluoroquinolone-resistant or extended-spectrum β-lactamase–producing *E. coli* of nonhospitalized persons has been described as an emerging phenomenon (*6**–**9*). Although the exact clinical implications of this phenomenon are not clearly established, colonization by these resistant strains is a public health threat at the community and hospital levels (*8**,**9*). The reasons for the increased prevalence of fecal carriage of these resistant *E. coli* strains by children from the studied areas are not clear. Data collected about household use of antimicrobial drugs excluded previous use of fluoroquinolones and expanded-spectrum cephalosporins (C. Kristiansson et al., unpub. data). The increased prevalence of resistant *E. coli* strains in preschool children most likely reflects increased exposure within a contaminated household setting, in the food chain, or both (*6**,**8**,**10*).

Our findings support the need to continue monitoring the evolution of resistance in commensal *E. coli*, to evaluate the effects of these important reservoirs of resistance genes distributed in the community, to investigate the epidemiologic relationship with clinical isolates, and to define the role of the food supply. Investigation into whether carriage of resistant strains in adults correlates with data on antimicrobial-drug use in hospitals and in the community would also be of interest.
